# Thermal Stability and Decomposition Kinetics of 1-Alkyl-2,3-Dimethylimidazolium Nitrate Ionic Liquids: TGA and DFT Study

**DOI:** 10.3390/ma14102560

**Published:** 2021-05-14

**Authors:** Jianwen Meng, Yong Pan, Fan Yang, Yanjun Wang, Zhongyu Zheng, Juncheng Jiang

**Affiliations:** 1 College of Safety Science and Engineering, Nanjing Tech University, Nanjing 211816, China; 201861201056@njtech.edu.cn (J.M.); 201961201040@njtech.edu.cn (F.Y.); yanjun.wang@njtech.edu.cn (Y.W.); zzy1951506459@163.com (Z.Z.); ypnjut@126.com (J.J.); 2 School of Environment and Safety Engineering, Changzhou University, Changzhou 213164, China

**Keywords:** ionic liquids, 1-alkyl-2,3-dimethylimidazolium nitrates, thermal hazard, kinetics analysis, density functional theory calculations

## Abstract

The thermal stability and decomposition kinetics analysis of 1-alkyl-2,3-dimethylimidazole nitrate ionic liquids with different alkyl chains (ethyl, butyl, hexyl, octyl and decyl) were investigated by using isothermal and nonisothermal thermogravimetric analysis combined with thermoanalytical kinetics calculations (Kissinger, Friedman and Flynn-Wall-Ozawa) and density functional theory (DFT) calculations. Isothermal experiments were performed in a nitrogen atmosphere at 240, 250, 260 and 270 °C. In addition, the nonisothermal experiments were carried out in nitrogen and air atmospheres from 30 to 600 °C with heating rates of 5, 10, 15, 20 and 25 °C/min. The results of two heating modes, three activation energy calculations and density functional theory calculations consistently showed that the thermal stability of 1-alkyl-2,3-dimethylimidazolium nitrate ionic liquids decreases with the increasing length of the alkyl chain of the substituent on the cation, and then the thermal hazard increases. This study could provide some guidance for the safety design and use of imidazolium nitrate ionic liquids for engineering.

## 1. Introduction

Ionic liquids (ILs), also known as room-temperature molten salt and nonaqueous organic molten salt, are a special kind of organic molten salt that is formed by the combination of organic cations and inorganic or organic anions [1,2]. They have different molecular symmetries and charge delocalization and present a molten state at room temperature [3]. Ionic liquids have been widely used in the fields of adsorption and electrochemistry. This is due to the designability of ionic liquids, which allows the target design of functionalized ionic liquids by varying the combination of anions and cations with different physicochemical properties according to specific applications [4,5,6]. In consequence, it is of great theoretical significance and practical value to investigate the thermal decomposition process of ionic liquids for the synthesis method of ionic liquids, the optimization of environmental parameters in the process of use and transportation, the safety design of fire and explosion prevention and the selection of safety facilities and equipment in the actual industrial process.

Although ionic liquids have a wider stable temperature range than other organic solvents, not all kinds of ionic liquids are stable [7,8,9]. This is because the thermal stability of ionic liquids is affected by the interaction between noncarbon atoms and carbon atoms and between noncarbon atoms and hydrogen bonds [10,11]. Meanwhile, it is also affected by the charge density of cations and the type of anions. In addition, the number and position of substituents on the cationic ring, the length and type of alkyl chain also play a decisive role in the thermal stability of ionic liquids [12,13].

For a long time, due to the lack of flash point temperature in the traditional sense, ionic liquids were not considered to be flammable and volatile, and the danger of ionic liquids was ignored [14,15,16]. Fox et al. experimentally studied some imidazole ionic liquids using TGA and a flash-ignition temperature tester and found that most of the studied ionic liquids showed obvious undercooling, and the degree of undercooling decreased with the increase in the number of carbon atoms in the substituent alkyl chain of cations [17]. Liu et al. demonstrated that the flammability of some imidazole ionic liquids is due to the flammability of their decomposition products by using a variety of experimental equipment [18,19,20]. For imidazole-containing ionic liquids with basic ([Cl]^−^) and weak coordination ([BF_4_]^−^, [NTF_2_]^−^) anions, the increase in the number of carbon atoms in the alkyl chain will increase the thermal risk. The increase in thermal hazard is due to the increased stability of carbon positive ions and carbon radicals when the number of carbon atoms in the alkyl chain increases [21,22,23].

At present, the traditional test is the basic method to evaluate the thermal hazard of ionic liquids. However, due to the high complexity and diversity of the structure of ionic liquids, it is necessary to study its structural characteristics and intermolecular forces [24,25,26]. Li et al. analyzed and calculated the hydrogen bonds, intermolecular forces, bond energies and activation energies of [Bmim][DBP] by using B3LYP/6–311++G (d,p) and M06–2X/6–311++G(d,p) levels of calculation [27]. Nie et al. used a combination of initio calculations, molecular dynamics simulations, Born–Haber cycle analysis and iso-chain reactions to find that all ion pairs can form strong hydrogen bonds, and the interaction is determined by the side-chain structure and functional groups of amino acid anions [28].

To date, no relevant studies on the thermal stability of the ionic liquids 1-alkyl-2,3-dimethylimidazolium nitrate class have been reported. Therefore, in this paper, thermogravimetry (TGA) was used to investigate the thermal stability of 1-alkyl-2,3-dimethylimidazolium nitrate under isothermal and nonisothermal conditions. At the same time, theoretical studies were conducted by combining the frontier molecular orbital theory with quantum chemical theory calculations. Experimental and theoretical studies on the thermal hazard of ionic liquids are of positive significance in guiding the design and safe application of new ionic liquids.

## 2. Experiments and Methods

### 2.1. Materials

In this study, five ionic liquids with 99% mass purity, namely 1-ethyl-2,3-dimethylimidazolium nitrate, 1-butyl-2,3-dimethylimidazolium nitrate, 1-hexyl-2,3-dimethylimidazolium nitrate, 1-octyl-2,3-dimethylimidazolium nitrate and 1-decyl-2,3-dimethylimidazole nitrate, were provided by the Lanzhou Institute of Chemical Physics, Chinese Academy of Sciences(Lanzhou, China). To avoid the influence of impure water on the experimental results, the samples were stored in a vacuum drying oven at 50 °C for one week before the experiments.

The detailed chemical information of the five ionic liquids is shown in Table 1.

### 2.2. Experimental Apparatus and Test Procedure

A TGA 3+110LF analyzer (METTLER-TOLEDO, Zurich, Switzerland) was used to test the thermal stability of the five ionic liquids. Samples (5.0–8.0 mg, 70 μL) were placed in crucibles made of Al_2_O_3_ and heated from 30 to 600 °C with heating ramp rates of 5, 10, 15, 20 and 25 °C/min. Furthermore, to compare the thermal stability and thermal effects under different atmospheres, the experiments were performed by purging the crucibles with a flow rate of 20 mL/min nitrogen and air. Isothermal experiments were performed at 240, 250, 260 and 270 °C in nitrogen atmosphere. The experimental results received various factors such as the heating rate, the furnace atmosphere and the sensitivity of the balance. It should be noted that to ensure the reliability of the experimental results, each TGA test group in the experimental study was repeated two times under the same conditions. Moreover, the final experimental data were averaged over three measurements.

### 2.3. Computational Methods

In this study, the initial structures of 1-alkyl-2,3-dimethylimidazole nitrate ionic liquids were constructed by GaussView 6.0 [29,30,31]. The molecular structures of [Emmim][NO_3_], [Bmmim][NO_3_], [Hmmim][NO_3_], [Ommim][NO_3_] and [Dmmim][NO_3_] were optimized by the density function theory (DFT) method accompanied with a 6–311++G(d,p) basis set. One of the important analyses for density functional theory calculations, front-line molecular orbital analysis, contains the highest occupied orbital HOMO and the lowest unoccupied empty orbital LUMO [32,33,34]. After optimization and vibrational analysis of the ionic liquids using the Multiwfn program, the HOMO and LUMO of the ionic liquids were obtained using the molecular orbital analysis function of Gauss view 6.0. The value of the highest occupied orbital HOMO size is influenced by the electron supply capacity, while the value of the lowest unoccupied empty orbital LUMO size is influenced by the electron capacity. The difference between the ∆E, the energy difference of the frontier orbitals, can not only determine the stability of the molecule but also indicate the reaction path of the molecule with other systems [35,36].

## 3. Results and Discussion

### 3.1. Short-Term Thermal Stability

Four characterization parameters obtained from TGA curves were chosen: the temperature value corresponding to the start of weight loss (*T_start_*), the temperature value corresponding to the maximum rate of weight loss (*T_peak_*), the temperature value corresponding to the intersection of the extension of the mass baseline at the very beginning and the tangent line at the *T_peak_* point (*T_onset_*) and the residual amount remaining at the end of the experiment to study the short-term thermal stability of [Emmim][NO_3_], [Bmmim][NO_3_], [Hmmim][NO_3_], [Ommim][NO_3_] and [Dmmim][NO_3_] [37,38]. Table 2 summarizes the above-mentioned important characteristic parameters of the five ionic liquids TGA curves in N_2_ atmosphere.

**Figure 1:**
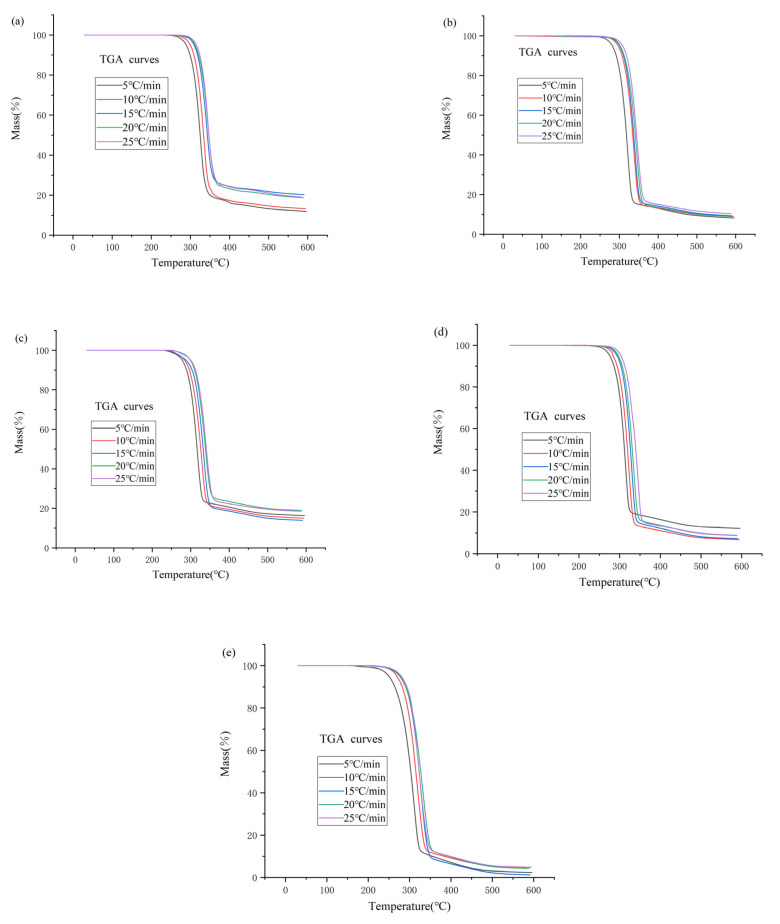
TGA curves of [Emmim][NO_3_] (**a**), [Bmmim][NO_3_] (**b**), [Hmmim][NO_3_] (**c**), [Ommim][NO_3_] (**d**) and [Dmmim][NO_3_] (**e**) with different heating rates.

The TGA curves of the1-alkyl-2,3-dimethylimidazole nitrate ionic liquids obtained at different heating rates are generally consistent in shape, but the TGA curves shift to the right as the heating rate increases, and the corresponding *T_start_*, *T_onset_* and *T_peak_* values become larger. In addition, as shown in Table 2, the higher the temperature increase on the thermal decomposition, the faster the reaction rate. The higher the heating rate, the shorter the process time for the ionic liquid to reach the required temperature, and then the overall thermal decomposition reaction process time will be shorter. The TGA curves of the thermal decomposition of 1-alkyl-2,3-dimethylimidazole nitrate ionic liquids in nitrogen atmosphere and at a heating rate of 5, 10, 15, 20 and 25 °C/min are shown in Figure 1.

As shown in Figure 1, the thermal decomposition reaction of the 1-alkyl-2,3-dimethylimidazole nitrate ionic liquids is a one-step reaction, and the slight weight loss at about 100 °C causes the curve to fluctuate slightly, which is due to the evaporation of a small amount of water in the molten ionic liquid at high temperature. Moreover, with the increase in the heating rate, the temperature value corresponding to the beginning of weight loss of ionic liquids becomes larger.

For the values of the thermal decomposition characteristic parameters *T_start_*, *T_onset_* and *T_peak_* corresponding to the five ionic liquids, all results show that: [Emmim][NO_3_] > [Bmmim][NO_3_] > [Hmmim][NO_3_] > [Ommim][NO_3_] > [Dmmim][NO_3_]. Under the same conditions, the experimentally obtained *T_onset_* values are greater than *T_start_* values, but the mass loss of ionic liquids already occurs below the *T_onset_* values, so the thermal stability of the ionic liquids class in this study cannot be indicated by the *T_onset_* values. Cao and Xue also concluded that *T_onset_* values overestimate the thermal stability of ionic liquids when they studied other classes of ionic liquids [39,40]. Therefore, in this study, the *T_start_* value will be used to evaluate the thermal stability of 1-alkyl-2,3-dimethylimidazole nitrate ionic liquids, and the thermal stability can be obtained in descending order: [Emmim][NO_3_] > [Bmmim][NO_3_] > [Hmmim][NO_3_] > [Ommim][NO_3_] > [Dmmim][NO_3_]. This experimental result can indicate that the thermal stability of this type of ionic liquid decreases with the increase of the number of carbon atoms of the alkyl chain of the substituent group on the cation, and it is more likely to undergo thermal decomposition reactions at high-temperature conditions, and the thermal hazard increases.

**Figure 2:**
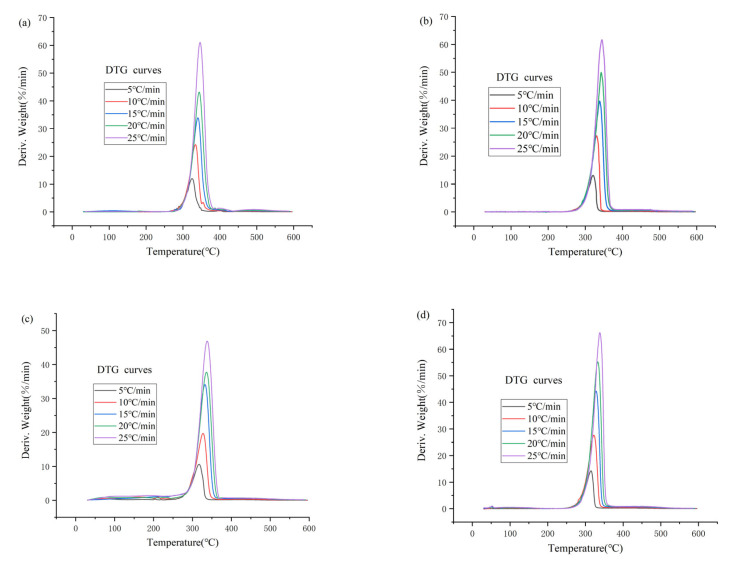

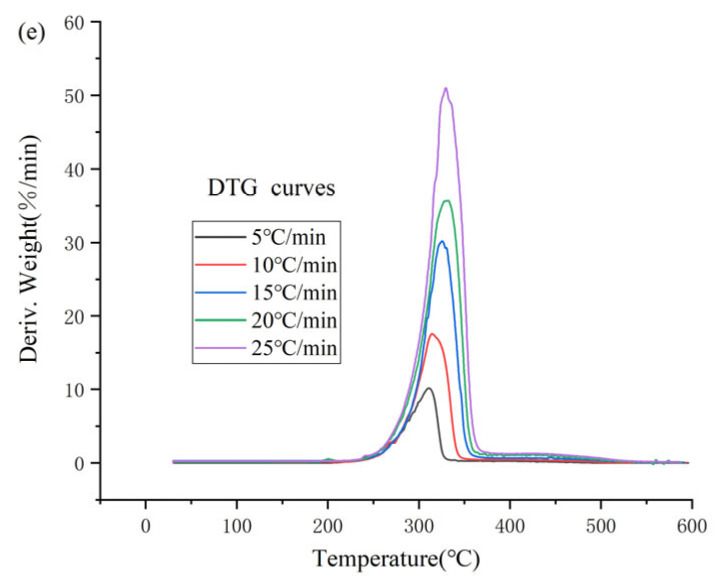
DTG curves of [Emmim][NO_3_] (**a**), [Bmmim][NO_3_] (**b**), [Hmmim][NO_3_] (**c**), [Ommim][NO_3_] (**d**) and [Dmmim][NO_3_] (**e**) with different heating rates.

As shown in Figure 2, it is obvious from the DTG curves that the thermal decomposition reaction process of all the five ionic liquids is one-step decomposition. The shapes of the DTG curves obtained for the 1-alkyl-2,3-dimethylimidazole nitrate ionic liquids at different temperature ramp rates of 5, 10, 15, 20 and 25 °C/min are generally consistent; the DTG curves shift to the right, and the peak widths become narrower with increasing ramp rates. This may be the result of the shortening of the thermal decomposition reaction time due to the increase of the temperature rise rate.

Since the one-step reaction process is similar, the one-step reaction is exemplified by 1-octyl-2,3-dimethylimidazole nitrate ([Hmmim][NO_3_]) at a ramp rate of 5 °C/min. Before 258 °C, the DTG curve showed slight fluctuations, which may be caused by the volatilization of some impurities contained in [Hmmim][NO_3_]. After 258 °C, the thermal decomposition reaction of [Hmmim][NO_3_] starts to occur. At about 298.27 °C, the degree of thermal decomposition of [Hmmim][NO_3_] gradually became larger, and the rate of thermal weight loss began to increase sharply and reached the maximum weight loss rate of DTG at 316.93 °C with 63.5% weight loss. Finally, from 400 to 600 °C, the decomposition of [Hmmim][NO_3_] is nearly complete with a residual mass of 16.26%. At the end of the experiment, it was found that the colors of the remaining material in the final crucible are all black because of the carbonization of the ionic liquid. Similarly, the thermal decomposition processes of the other four imidazole nitrate ionic liquids can be obtained.

**Figure 3:**
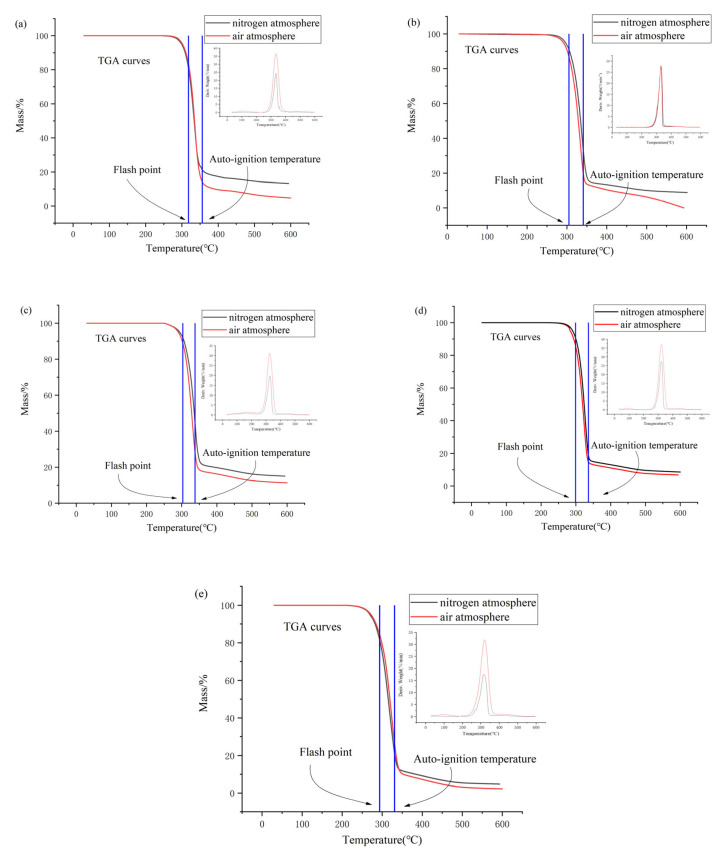
TGA–DTG curves of [Emmim][NO_3_] (**a**), [Bmmim][NO_3_] (**b**), [Hmmim][NO_3_] (**c**), [Ommim][NO_3_] (**d**) and [Dmmim][NO_3_] (**e**).

To better fit the actual industrial ionic liquids fire and explosion-proof intrinsically safe design and the selection of safety facilities and equipment, other conditions are the same as the nitrogen atmosphere experimental conditions. A temperature rate of 10 °C/min in the air atmosphere conditions of 1-alkyl-2,3-dimethylimidazole nitrate ionic liquids TGA experiments was used, and the experimental results and nitrogen atmosphere experimental results were compared. The TGA–DTG curve graph is shown in Figure 3, where the autoignition temperature and flash point temperature data are available from the authors’ previous studies [41]. The autoignition temperature and flash point temperature data are shown in Table 3.

According to the TGA curves in air atmosphere, the characteristic values of thermal decomposition *T_start_* for [Emmim][NO_3_], [Bmmim][NO_3_], [Hmmim][NO_3_], [Ommim][NO_3_] and [Dmmim][NO_3_] in air atmosphere conditions can be obtained as 276.63, 274.68, 268.93 260.45 and 229.83 °C, respectively. In agreement with the results for nitrogen atmosphere, the order of the initial decomposition temperatures is also [Emmim][NO_3_] > [Bmmim][NO_3_] > [Hmmim][NO_3_] > [Ommim][NO_3_] > [Dmmim][NO_3_], indicating that the thermal stability of this type of ionic liquids under both nitrogen and air conditions decreases with the increase in the number of carbon atoms of the alkyl chain of substituents on the cation. The *T_onset_* are 312.13, 310.23, 300.89, 298.36 and 286.25 °C, and the *T_peak_* are 332.46, 330.68, 325.10, 319.72 and 313.26 °C. The final residuals are 4.70, 0, 11.32, 8.58 and 2.22% for [Emmim][NO_3_], [Bmmim][NO_3_], [Hmmim][NO_3_], [Ommim][NO_3_] and [Dmmim][NO_3_], respectively.

As shown in Table 2 and Table 3 and Figure 3, both the nitrogen atmosphere and air atmosphere conditions showed that *T_start_* is less than *T_FP_*, and *T_FP_* is less than *T_AIT_*. Therefore, the main reason for the flash combustion and autoignition of 1-alkyl-2,3-dimethylimidazole nitrate ionic liquids could be attributed to the ionic liquids themselves for the thermal decomposition, which produces flammable or combustible substances.

It is also possible to analyze the differences in the experimental results obtained between nitrogen atmosphere and air atmosphere. By taking 1-octyl-2,3-dimethylimidazole nitrate ([Hmmim][NO_3_]) as an example, the *T_start_*, *T_onset_*, *T_peak_* and final residues of [Hmmim][NO_3_] in air atmosphere are smaller than the values in nitrogen atmosphere, and the peak height of the DTG curve is higher. This is due to the fact that under conditions with high temperature and air atmosphere, the ionic liquids and the decomposition products undergo not only decomposition reactions but also oxidation reactions, resulting in the advancement of the thermal decomposition reaction and the increase in the reaction rate, as well as the increase in the reaction time and the decrease in the final residual black carbide in the crucible. The same conclusion can be obtained when comparing the other four ionic liquids. This result also indicates that in the 1-alkyl-2,3-dimethylimidazole nitrate ionic liquids are more susceptible to thermal decomposition at high temperatures and in the presence of air atmosphere.

### 3.2. Long-Term Thermal Stability

Considering the perspective of practical industrial applications, ionic liquids may need to be applied in high-temperature environments for long periods of time. Under such high loading conditions, both *T_start_* and *T_onset_* are no longer suitable for characterizing the long-term thermal stability of ionic liquids. As one of the purposes of this study is to investigate the long-term and short-term thermal stability of 1-alkyl-2,3-dimethylimidazole nitrate ionic liquids, in this study, the long-term thermal stability was tested by isothermal measurements at different temperature intervals around *T_start_* for the five substances selected.

**Figure 4:**
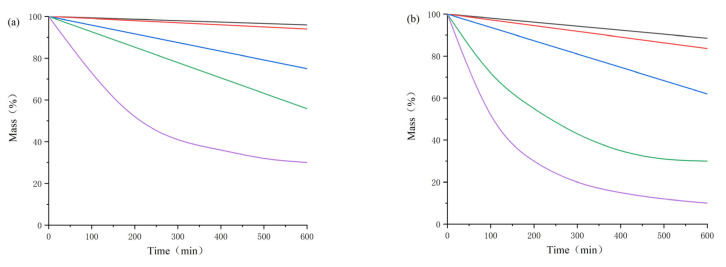

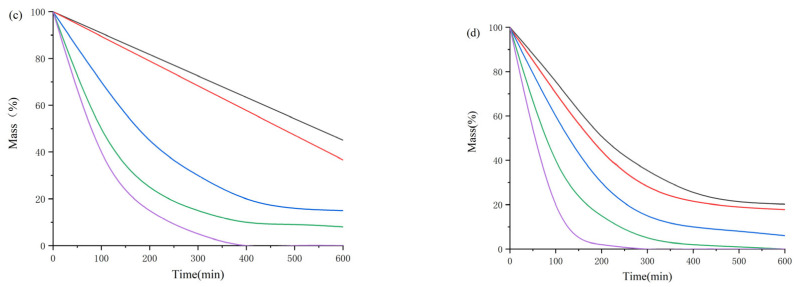
Isothermal TGA curves of five ionic liquids at 240 °C (**a**), 250 °C (**b**), 260 °C (**c**) and 270 °C (**d**). ([Emmim][NO_3_], [Bmmim][NO_3_], [Hmmim][NO_3_], [Ommim][NO_3_] and [Dmmim][NO_3_] are represented by black, red, blue, green and purple curves, respectively).

Figure 4 shows the results of isothermal TGA experiments of five ionic liquids at different temperature intervals (270–240 °C with a temperature interval of 10 °C). It can be seen that with the increase in the number of carbon atoms of the alkyl chain of substituents on the cation and the increase in temperature, the weight loss of 1-alkyl-2,3-dimethylimidazole nitrate ionic liquids is more obvious after 10 h of constant temperature, which indicates that the magnitude of the long-term thermal stability of the 1-alkyl-2,3-dimethylimidazole nitrate ionic liquids is [Emmim][NO_3_] > [Bmmim][NO_3_] > [Hmmim][NO_3_] > [Ommim][NO_3_] > [Dmmim][NO_3_], which is consistent with the short-term thermal stability law.

Compared with the ionic liquids thermal decomposition temperature value in Table 2, the *T_start_* value is much higher than the long-term thermal decomposition temperature, indicating that the long-term thermal stability of such ionic liquids is significantly lower than the short-term thermal stability. Therefore, for the long-term use of ionic liquids, the short-term thermal stability characteristics parameters cannot be used as a reference basis. It needs to strengthen the temperature monitoring to avoid accidents.

### 3.3. Kinetics of Decomposition

Activation energy is one of the basic parameters to evaluate the thermal hazard of ionic liquids, but there are differences in the magnitude of the activation energy values calculated by different isoconversion rate methods at nonisothermal temperatures. In order to verify the accuracy of the activation energy values obtained for the 1-alkyl-2,3-dimethylimidazole nitrate ionic liquids, this study used three different calculation methods: Kissinger, Friedman and the Flynn–Wall–Ozawa (FWO) method based on the nonisothermal thermogravimetric TGA–DTG curve data to calculate the activation energy of *E_a_* [42,43,44,45].

The equation for the FWO method is Equation (1), the equation for the Kissinger method is Equations (2) and (3), and the equation for the Friedman method is Equation (4).

(1) $$\mathrm{lg}\left(\beta \right)=\mathrm{lg}\left[\frac{AE_{a}}{RG\left(\alpha \right)}\right]-2.315-0.4567\frac{E_{a}}{RT}$$

(2) $$\ln\frac{\beta }{T^{2}}=\ln\left[\frac{AR}{G\left(\alpha \right)E_{a}}\right]-\frac{E_{a}}{RT}$$

(3) $$\ln\frac{\beta }{T_{p}^{2}}=\ln\left(\frac{AR}{E_{a}}\right)-\frac{E_{a}}{RT_{p}}$$

(4) $$\ln\frac{\beta d_{\alpha }}{d_{T}}=\ln\left[Af\left(\alpha \right)\right]-\frac{E_{a}}{RT}$$

**Figure 5:**
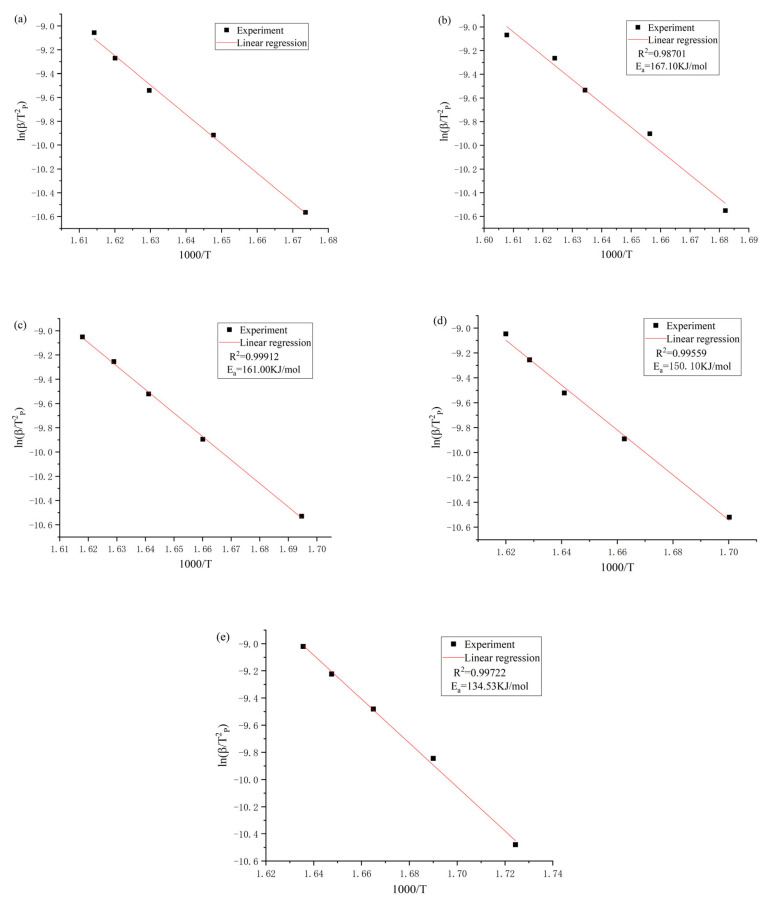
Fitting results of the Kissinger method for [Emmim][NO_3_] (**a**), [Bmmim][NO_3_] (**b**), [Hmmim][NO_3_] (**c**), [Ommim][NO_3_] (**d**) and [Dmmim][NO_3_] (**e**).

The activation energies of [Emmim][NO_3_], [Bmmim][NO_3_], [Hmmim][NO_3_], [Ommim][NO_3_] and [Dmmim][NO_3_] can be obtained by Equation (3) of the Kissinger method and the fitting linear slope calculation as 205.00 ± 8.17, 167.10 ± 9.05, 161.00 ± 6.80, 150.10 ± 7.68 and 134.53 ± 6.95 kJ/mol, and the corresponding R^2^ of the fits were 0.99582, 0.98701, 0.99912, 0.99559 and 0.99722, respectively. The results of the fitted relationship diagram are shown in Figure 5.

**Figure 6:**
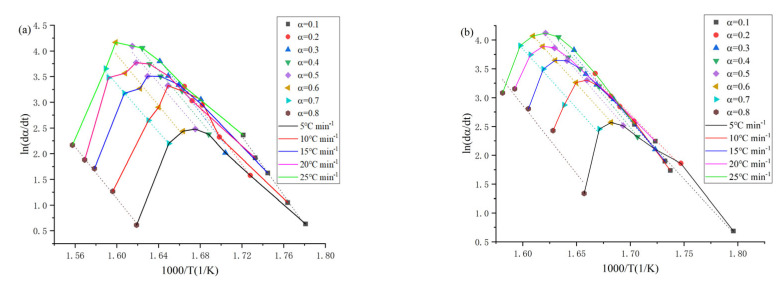

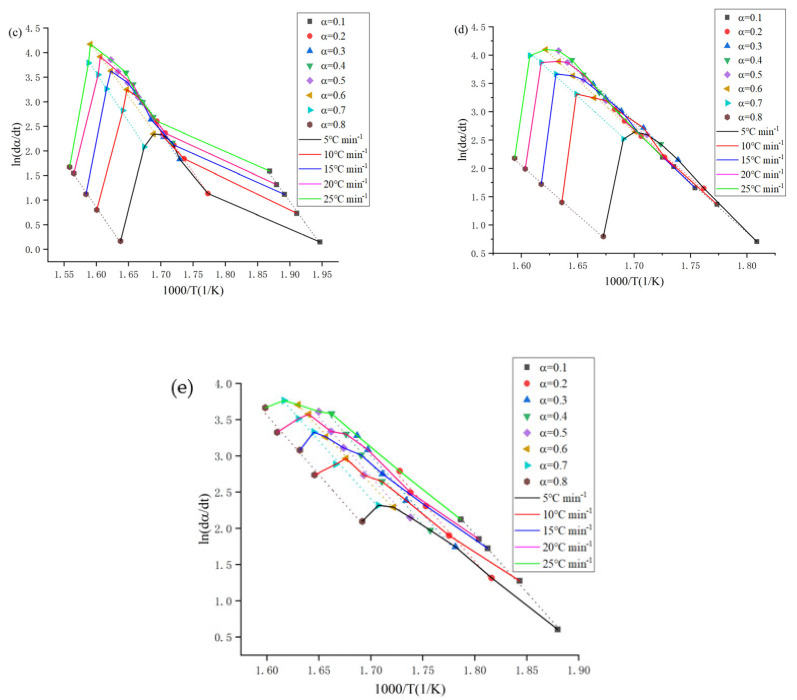
Fitting results of the Friedman method for [Emmim][NO_3_] (**a**), [Bmmim][NO_3_] (**b**), [Hmmim][NO_3_] (**c**), [Ommim][NO_3_] (**d**) and [Dmmim][NO_3_] (**e**).

Figure 6 shows the activation energy curves of the 1-alkyl-2,3-dimethylimidazole nitrate ionic liquids obtained by the Friedman method, and from the activation energy graphs, the trend of each ionic liquids is basically the same. Combined with the analysis of the activation energy values obtained by Friedman and FWO method in Table 4 and Table 5, it can be found that each ionic liquid has a small difference between the different conversion rates, and it can be inferred that [Emmim][NO_3_], [Bmmim][NO_3_], [Hmmim][NO_3_], [Ommim][NO_3_] and [Dmmim][NO_3_] all contain only one thermal decomposition reaction process.

**Figure 7:**
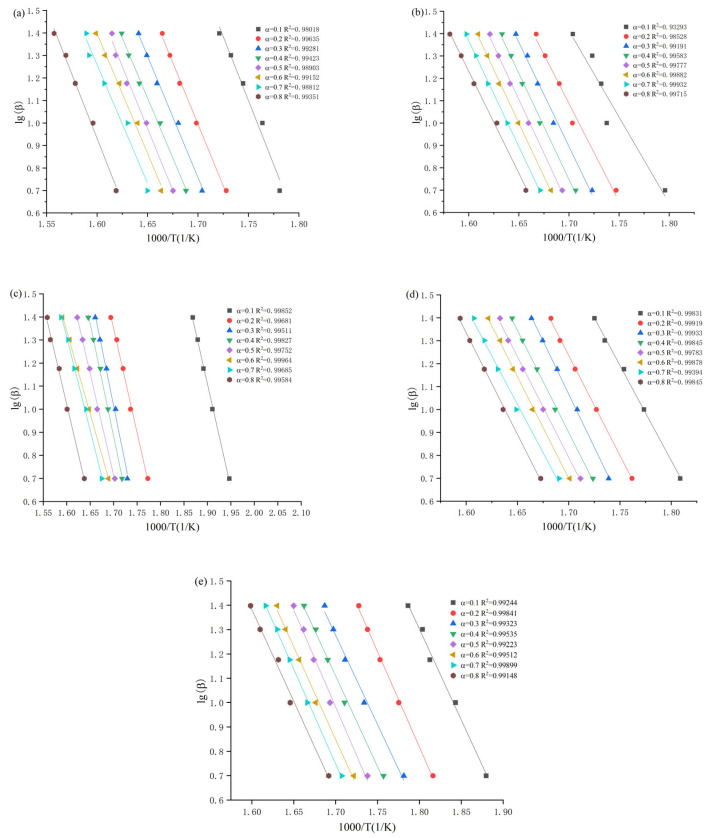
Fitting results of the Flynn–Wall–Ozawa method for [Emmim][NO_3_] (**a**), [Bmmim][NO_3_] (**b**), [Hmmim][NO_3_] (**c**), [Ommim][NO_3_] (**d**) and [Dmmim][NO_3_] (**e**).

Based on the results of TGA curves for different heating rates of 5, 10, 15, 20 and 25 °C/min in nitrogen atmosphere, the experimental results of conversion α in the interval range of 0.1–0.8 with each group interval of 0.1 were selected as the base datausing MATLAB 2019b (MathWorks company, Natick, American) program, and the mathematical least-squares method was introduced to bring in the experimental data for fitting to plot the relationship between the *Y* coordinate of $\ln\left(\beta \right)\;$and the *X* coordinate of 1000/T, as shown in Figure 7. The average activation energies of the 1-alkyl-2,3-dimethylimidazole nitrate ionic liquids were obtained as 202.43 ± 3.38, 166.16 ± 2.83, 161.74 ± 3.15, 150.19 ± 4.03 and 135.03 ± 3.73 kJ/mol, respectively, with R^2^ above 0.99 by Equation (1), and the results are similar to those calculated by the Kissinger and Friedman methods.

The difference in activation energy between the three calculation methods is small, and the fit of each is above 0.98. Therefore, the activation energies of the 1-alkyl-2,3-dimethylimidazole nitrate ionic liquids should be taken as the average of the Kissinger, Friedman, and FWO methods, and the activation energies of [Emmim][NO_3_], [Bmmim][NO_3_], [Hmmim][NO_3_], [Ommim][NO_3_] and [Dmmim][NO_3_] are 204.45 ± 5.80, 169.95 ± 6.08, 161.55 ± 4.97, 150.81 ± 5.83 and 134.75 ± 6.12 kJ/mol. As shown in Table 6, the magnitude of the thermal decomposition hazard of the 1-alkyl-2,3-dimethylimidazole nitrate ionic liquids is: [Emmim][NO_3_] < [Bmmim][NO_3_] < [Hmmim][NO_3_] < [Ommim][NO_3_] < [Dmmim][NO_3_].

### 3.4. Front-Line Molecular Orbital Analysis

**Figure 8:**
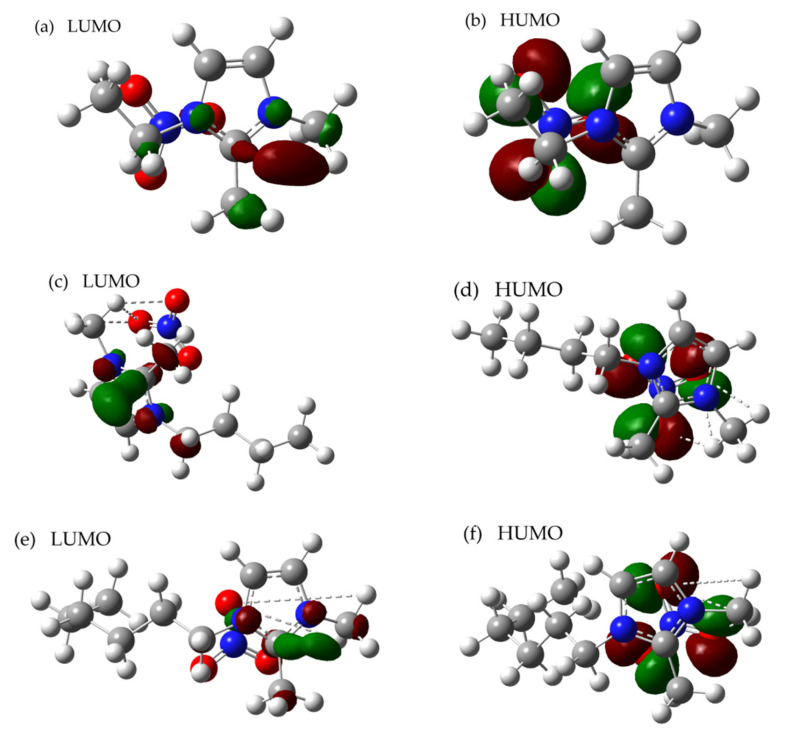

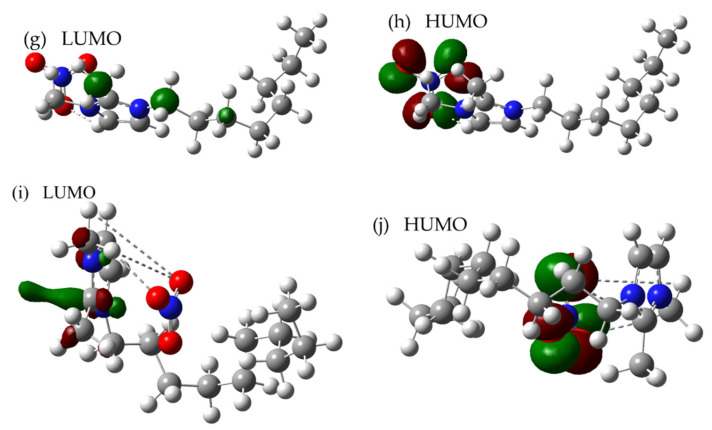
The results of the frontier molecular orbital for [Emmim][NO_3_] (**a**,**b**), [Bmmim][NO_3_] (**c**,**d**), [Hmmim][NO_3_] (**e**,**f**), [Ommim][NO_3_] (**g**,**h**) and [Dmmim][NO_3_] (**i**,**j**).

As can be seen from Figure 8, the LUMO electron of [Emmim][NO_3_], [Bmmim][NO_3_], [Hmmim][NO_3_], [Ommim][NO_3_] and [Dmmim][NO_3_] are all distributed in the nitro portion, and the calculations indicate that this type of ionic liquid differs from most ionic liquids in that the anion nitro portion of the anion pair contains the highest electron density, causing a proton to react first with the anion in the anion–cation ion pair. On the contrary, the LUMO electron cloud mainly concentrates on imidazole, and this phenomenon indicates that if the electron leap from HOMO to LUMO occurs, the electrons of the nitrate ion in the ionic liquid will preferentially transfer intermolecular charge to the imidazole ring, so the imidazole ring is less prone to chemical bond breakage. The cation will preferentially break the chemical bond over the anion, which is one of the reasons why the overall thermal stability of this type of ionic liquid is better than other types.

Meanwhile, it can be obtained from the Table 7 that the energy difference is: [Emmim][NO_3_] > [Bmmim][NO_3_] > [Hmmim][NO_3_] > [Ommim][NO_3_] > [Dmmim][NO_3_]. The molecular structure stability order is: [Emmim][NO_3_] > [Bmmim][NO_3_] > [Hmmim][NO_3_] > [Ommim][NO_3_] > [Dmmim][NO_3_].

The results of density functional theory calculations and TGA experimental are consistent: the thermal stability of ionic liquids of this type decreases and the thermal hazard increases as the number of carbon atoms in the alkyl chain of substituents on the cation increases. Finally, the results of the study are consistent with the trend of the change in thermal stability of imidazolium-based ionic liquids containing [Cl]^−^, [BF_4_]^−^, [PF_6_]^−^ and [NTF_2_]^−^ anions with the change in cationic alkyl chain length.

## 4. Conclusions

Isothermal and nonisothermal thermogravimetric analyses were performed to assess the thermal stability of 1-alkyl-2,3-dimethylimidazolium nitrate ionic liquids with different alkyl chains (ethyl, propyl, butyl, hexyl and decyl) pairing with the nitrate anion. The length of the alkyl group on the imidazolium ring was a strong indicator of the characteristic parameter values of thermal stability such as *T_start_*, *T_onset_*, *T_peak_* and residual amount. TGA–DTG curves for 1-alkyl-2,3-dimethylimidazole nitrate ionic liquids in nitrogen and air atmospheres with different heating rates demonstrated that it was a one-step reaction for their thermal decomposition in both nitrogen and air atmospheres.

Nonisothermal and isothermal thermogravimetric experimental results showed that the short-term thermal stability of 1-alkyl-2,3-dimethylimidazolium nitrate ionic liquids and long-term thermal stability are the same. The thermal stability of 1-alkyl-2,3-dimethylimidazolium nitrate ionic liquids was [Emmim][NO_3_] > [Bmmim][NO_3_] > [Hmmim][NO_3_] > [Ommim][NO_3_] > [Dmmim][NO_3_], which indicated that the thermal stability of 1-alkyl-2,3-dimethylimidazole nitrate ionic liquids decreases with the increase in the substituent alkyl chain carbon atom number on the cation. Furthermore, the long-term thermal stability of 1-alkyl-2,3-dimethylimidazolium nitrate ionic liquids was significantly lower than the short-term thermal stability.

Based on the TGA–DTG curves data under nitrogen atmosphere, the results of the activation energy calculations obtained by using the Kissinger, Friedman and FWO methods were similar, with R^2^ being above 0.98. The average activation energy for thermal decomposition of 1-alkyl-2,3-dimethylimidazole nitrate ionic liquids were 204.45 ± 5.80, 169.95 ± 6.08, 161.55 ± 4.97, 150.81 ± 5.83 and 134.75 ± 6.12 kJ/mol for [Emmim][NO_3_], [Bmmim][NO_3_], [Hmmim][NO_3_], [Ommim][NO_3_] and [Dmmim][NO_3_], respectively.

The thermal stability relationships of the 1-alkyl-2,3-dimethylimidazole nitrate ionic liquids were obtained from the frontier molecular orbital theory, and the results were compared with those of the experimental study of thermal hazards to verify the consistency and reliability of the theoretical calculations and experimental results. The molecular structure stability was [Emmim][NO_3_] > [Bmmim][NO_3_] > [Hmmim][NO_3_] > [Ommim][NO_3_] > [Dmmim][NO_3_], which was inconsistent with those of experimental studies on thermal stability.

This study could provide some guidance for the safety design and use of imidazolium nitrate ionic liquids in practical engineering applications.

## Figures and Tables

**Table 1:** Chemical information for five ionic liquids.

Chemical Name	Abbreviation	Formula	Weight (g/mol)	Structural Formula
1-ethyl-2,3-dimethylimidazolium nitrate	[Emmim][NO_3_]	C_7_H_13_O_3_N_3_	187.20	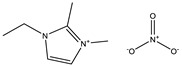
1-butyl-2,3-dimethylimidazolium nitrate	[Bmmim][NO_3_]	C_9_H_17_O_3_N_3_	215.25	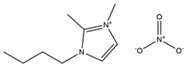
1-hexyl-2,3-dimethylimidazolium nitrate	[Hmmim][NO_3_]	C_11_H_21_O_3_N_3_	243.30	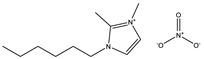
1-octyl-2,3-dimethylimidazolium nitrate	[Ommim][NO_3_]	C_13_H_25_O_3_N_3_	271.36	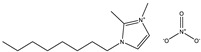
1-decyl-2,3-dimethylimidazolium nitrate	[Dmmim][NO_3_]	C_15_H_29_O_3_N_3_	299.41	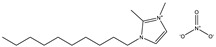

**Table 2:** Obtained mass loss parameters for five ionic liquids by TGA.

Chemical Name	*β* (°C/min)	Temperature (°C)			Residue (%)
		*T_start_*	*T_onset_*	*T_peak_*	
[Emmim][NO_3_]	5	276.10 ± 1.25	307.99 ± 0.99	324.36 ± 2.38	11.93 ± 0.92
	10	278.68 ± 2.36	313.99 ± 1.37	333.74 ± 3.02	13.27 ± 1.19
	15	287.81 ± 1.88	321.78 ± 0.69	340.46 ± 3.22	19.36 ± 0.88
	20	294.56 ± 1.06	324.58 ± 2.56	344.10 ± 1.54	18.77 ± 1.02
	25	297.02 ± 2.03	327.61 ± 2.67	346.35 ± 1.61	19.13 ± 1.21
[Bmmim][NO_3_]	5	274.78 ± 1.95	304.77 ± 0.65	321.37 ± 3.55	7.82 ± 0.61
	10	275.50 ± 2.08	311.96 ± 1.02	330.58 ± 3.79	8.09 ± 0.41
	15	277.42 ± 1.55	319.53 ± 0.54	338.72 ± 1.69	8.47 ± 0.56
	20	279.11 ± 1.56	322.96 ± 2.11	342.60 ± 2.34	8.07 ± 0.38
	25	280.17 ± 0.98	323.26 ± 2.01	344.84 ± 1.02	9.87 ± 0.33
[Hmmim][NO_3_]	5	258.43 ± 2.33	298.27 ± 0.94	316.93 ± 3.20	16.26 ± 0.16
	10	269.72 ± 1.63	303.36 ± 1.33	327.23 ± 2.81	14.95 ± 0.80
	15	275.11 ± 1.92	308.52 ± 1.89	332.19 ± 1.99	13.86 ± 1.22
	20	277.35 ± 2.06	315.00 ± 1.57	335.74 ± 3.02	18.93 ± 1.20
	25	279.02 ± 1.12	317.19 ± 2.01	337.91 ± 2.00	18.45 ± 1.90
[Ommim][NO_3_]	5	253.08 ± 1.52	294.73 ± 1.97	315.00 ± 2.10	12.12 ± 1.11
	10	265.43 ± 0.90	301.39 ± 1.60	322.33 ± 1.87	6.79 ± 0.69
	15	271.34 ± 1.42	309.34 ± 2.30	328.24 ± 1.93	7.29 ± 0.80
	20	274.38 ± 1.26	312.66 ± 2.61	332.90 ± 2.35	8.79 ± 1.00
	25	275.18 ± 2.03	318.02 ± 2.15	338.13 ± 2.08	8.93 ± 0.91
[Dmmim][NO_3_]	5	214.49 ± 1.50	278.56 ± 0.69	310.76 ± 1.24	2.34 ± 0.28
	10	232.85 ± 1.19	290.30 ± 0.90	314.57 ± 1.60	4.71 ± 0.61
	15	243.17 ± 2.01	299.54 ± 0.58	325.46 ± 1.18	1.27 ± 0.36
	20	245.94 ± 1.66	300.30 ± 1.27	331.83 ± 2.01	4.23 ± 0.58
	25	248.34 ± 2.34	302.33 ± 1.33	332.25 ± 1.65	4.73 ± 0.47

**Table 3:** The data of autoignition temperature and flash point temperature.

Name	*T_FP_*/°C	*T_AIT_*/°C	The Ignition Delay Time/s
[Emmim][NO_3_]	318.1 ± 0.9	356 ± 17.8	10.8
[Bmmim][NO_3_]	305.7 ± 2.3	341 ± 17.1	8.6
[Hmmim][NO_3_]	301.3 ± 1.9	338 ± 16.9	8.2
[Ommim][NO_3_]	298.9 ± 1.5	336 ± 16.8	7.6
[Dmmim][NO_3_]	293.5 ± 2.1	331 ± 16.6	6.2

**Table 4:** The activation energy obtained by the Friedman method for thermal decomposition of the 1-alkyl-2,3-dimethylimidazole nitrate ionic liquids.

α	*E_a_* (kJ/mol)				
	[Emmim][NO_3_]	[Bmmim][NO_3_]	[Hmmim][NO_3_]	[Ommim][NO_3_]	[Dmmim][NO_3_]
0.1	212.41 ± 5.90	169.04 ± 4.98	151.48 ± 4.31	147.78 ± 4.92	133.98 ± 7.33
0.2	211.21 ± 6.22	157.66 ± 4.68	154.01 ± 4.88	153.42 ± 5.71	134.74 ± 8.56
0.3	217.62 ± 4.89	180.49 ± 7.52	164.42 ± 5.50	152.75 ± 5.80	134.18 ± 7.52
0.4	202.71 ± 5.81	186.80 ± 6.31	164.43 ± 4.79	154.75 ± 5.29	140.18 ± 6.99
0.5	196.86 ± 7.02	179.56 ± 8.01	156.17 ± 4.57	154.25 ± 6.44	135.83 ± 8.34
0.6	204.48 ± 6.09	171.43 ± 5.70	153.29 ± 5.72	153.55 ± 5.72	129.46 ± 8.00
0.7	192.83 ± 5.16	171.20 ± 6.58	164.36 ± 5.09	150.50 ± 6.00	133.50 ± 7.46
0.8	209.21 ± 5.74	196.55 ± 7.05	159.47 ± 4.90	153.77 ± 6.35	135.87 ± 7.29
Average	205.91 ± 5.85	176.59 ± 6.35	161.91 ± 4.97	152.14 ± 5.78	134.69 ± 7.69

**Table 5:** The activation energy obtained by the Flynn–Wall–Ozawa method for thermal decomposition of the 1-alkyl-2,3-dimethylimidazole nitrate ionic liquids.

α	*E_a_* (kJ/mol)				
	[Emmim][NO_3_]	[Bmmim][NO_3_]	[Hmmim][NO_3_]	[Ommim][NO_3_]	[Dmmim][NO_3_]
0.1	207.10 ± 3.45	140.63 ± 2.88	163.76 ± 3.12	151.03 ± 3.62	135.94 ± 3.68
0.2	199.47 ± 2.99	159.66 ± 2.31	164.27 ± 3.40	140.75 ± 4.55	135.44 ± 3.51
0.3	196.65 ± 3.06	169.39 ± 3.55	164.59 ± 2.80	150.53 ± 4.23	133.44 ± 4.66
0.4	212.02 ± 3.58	172.73 ± 2.70	177.84 ± 3.91	143.79 ± 3.71	135.79 ± 3.01
0.5	200.15 ± 2.69	174.94 ± 2.65	160.59 ± 3.48	159.87 ± 2.99	135.96 ± 3.80
0.6	197.14 ± 3.41	173.89 ± 1.88	148.73 ± 2.66	143.33 ± 3.40	129.44 ± 3.59
0.7	198.80 ± 4.12	172.24 ± 3.97	147.35 ± 2.17	153.52 ± 4.81	132.68 ± 4.09
0.8	208.15 ± 3.71	165.77 ± 2.69	158.32 ± 3.64	143.23 ± 4.93	137.26 ± 3.46
Average	202.43 ± 3.38	166.16 ± 2.83	161.74 ± 3.15	150.19 ± 4.03	135.03 ± 3.73

**Table 6:** The activation energy of the 1-alkyl-2,3-dimethylimidazole nitrate ionic liquids.

Method	*E_a_* (kJ/mol)				
	[Emmim][NO_3_]	[Bmmim][NO_3_]	[Hmmim][NO_3_]	[Ommim][NO_3_]	[Dmmim][NO_3_]
Kissinger	205.00 ± 8.17	167.10 ± 9.05	161.00 ± 6.80	150.10 ± 7.68	134.53 ± 6.95
Friedman	205.91 ± 5.85	176.59 ± 6.35	161.91 ± 4.97	152.14 ± 5.78	134.69 ± 7.69
FWO	202.43 ± 3.38	166.16 ± 2.83	161.74 ± 3.15	150.19 ± 4.03	135.03 ± 3.73
Average	204.45 ± 5.80	169.95 ± 6.08	161.55 ± 4.97	150.81 ± 5.83	134.75 ± 6.12

**Table 7:** The value results of the frontier molecular orbital.

	Energy (ev)				
	[Emmim][NO_3_]	[Bmmim][NO_3_]	[Hmmim][NO_3_]	[Ommim][NO_3_]	[Dmmim][NO_3_]
the LUMO energy	−0.00956	−0.01238	−0.01003	−0.02401	−0.02130
the HUMO energy	−0.27919	−0.27624	−0.26490	−0.26323	−0.25862
∆E	0.26963	−0.26323	−0.25862	0.23922	0.23732

## Data Availability

The data presented in this study are available on request from the corresponding author.

## Nomenclature

*T_AIT_*	The autoignition temperature (°C)
*T_FP_*	The flash point temperature (°C)
*T_start_*	Start temperature of decomposition (°C)
*T_peak_*	Peak temperature of decomposition (°C)
*T_onset_*	Onset temperature of decomposition (°C)
T	Reaction temperature (°C)
*E_a_*	Apparent activation energy (kJ/mol)
$G\left(\alpha \right)$	The integral mechanism function (dimensionless)
$f\left(\alpha \right)$	The most probable kinetic function (dimensionless)
A	Pre-exponential factor (min^−1^)
R	Universal gas constant (8.314 J/mol K)
α	Degree of conversion (dimensionless)
β	Heating rates (°C /min)
